# The *MP65 gene *is required for cell wall integrity, adherence to epithelial cells and biofilm formation in *Candida albicans*

**DOI:** 10.1186/1471-2180-11-106

**Published:** 2011-05-16

**Authors:** Silvia Sandini, Annarita Stringaro, Silvia Arancia, Marisa Colone, Francesca Mondello, Susanna Murtas, Antonietta Girolamo, Nicolina Mastrangelo, Flavia De Bernardis

**Affiliations:** 1 Department of Infectious, Parasitic and Immuno-mediated Diseases, Istituto Superiore di Sanità, Viale Regina Elena 299, Rome, 00161, Italy; 2 Department of Technology and Health, Istituto Superiore di Sanità, Viale Regina Elena 299, Rome, 00161, Italy; 3 Center for Immunobiologicals Research and Evaluation, Istituto Superiore di Sanità, Viale Regina Elena 299, Rome, 00161, Italy

## Abstract

**Background:**

The *MP65 *gene of *Candida albicans *(orf19.1779) encodes a putative β-glucanase mannoprotein of 65 kDa, which plays a main role in a host-fungus relationship, morphogenesis and pathogenicity. In this study, we performed an extensive analysis of a *mp65Δ *mutant to assess the role of this protein in cell wall integrity, adherence to epithelial cells and biofilm formation.

**Results:**

The *mp65Δ *mutant showed a high sensitivity to a range of cell wall-perturbing and degrading agents, especially Congo red, which induced morphological changes such as swelling, clumping and formation of hyphae. The *mp65Δ *mutant showed an activation of two MAPKs (Mkc1p and Cek1p), a high level of expression of two stress-related genes (DDR48 and *SOD5*), and a modulated expression of β-glucan epitopes, but no gross changes in cell wall polysaccharide composition. Interestingly, the *mp65Δ *mutant displayed a marked reduction in adhesion to BEC and Caco-2 cells and severe defects in biofilm formation when compared to the wild type. All of the mentioned properties were totally or partially recovered in a revertant strain, demonstrating the specificity of gene deletion.

**Conclusions:**

We demonstrate that the *MP65 *gene of *Candida albicans *plays a significant role in maintaining cell wall integrity, as well as in adherence to epithelia and biofilm formation, which are major virulence attributes of this fungus.

## Background

*Candida albicans *is both a commensal and a pathogenic yeast, which is responsible for severe infections in humans, particularly in immunocompromised persons, such as AIDS and cancer patients, diabetics, newborns and the elderly [1,2]. Although several anti-*Candida *agents are currently available, such as amphotericin B, azoles and echinocandins, there is clearly a need for new specific anti-fungal agents and drug-targets [3]. The cell wall of *C. albicans *is an essential organelle that helps to withstand osmotic pressure and determines the shape of the cell. The cell wall is a plastic and dynamic structure, whose macromolecular composition, molecular organization and thickness can greatly vary depending on environmental conditions. The cell wall construction is also tightly controlled in space and time by many genes [4]. Within a host-parasite relationship, the cell wall of *C. albicans *lies at the crossroads of pathogenicity and therapeutics. It contributes to pathogenicity through adherence and invasion, and it is the target of both pharmacological and immunological antifungal therapy [5,6]. The cell wall comprises two main layers. The inner layer consists of a network of β1,3-glucan molecules, accounting for approximately 40% of the cell-wall mass, to which β1,6-glucan (about 20%) and chitin (2-4%) are covalently attached [7]. The outer layer is composed of a dense layer of mannoproteins, termed "cell wall proteins" (CWP), which account for 35-40% of the cell-wall mass. Based on their linkage to other cell wall polysaccharides, two classes of CWPs can be distinguished. One class, which constitutes the majority of the CWPs, consists of CWPs that are covalently linked to β1,6-glucan via a remnant of a GPI anchor [8,9]. The other class consists of the so-called "alkali sensitive linkage" (ASL)-CWPs, which are covalently linked to the β1,3-glucan network (without an interconnecting β1,6-glucan molecule) through an unknown linkage that is sensitive to mild alkaline conditions [10]. The best-described ASL-CWPs are the family of Pir-proteins (proteins with internal repeats). Pir-proteins are thought to be pre-proteins that are processed at Kex2 endoprotease recognition sites [11]; the N-terminal part of mature proteins contains conserved internal tandem repeats, and the C-terminal half shares a high sequence similarity including four conserved cysteines.

The *MP65 *gene encodes a cell wall mannoprotein (Mp65p) of *C. albicans*. In a previous study [12-14], our research group identified, generated, and intensely studied native and recombinant forms of Mp65p and found that it is a major target of immune response in humans and mice [15-17]; we also found that Mp65p is a critical determinant of pathogenicity in experimental models of systemic infection in mice and vaginal infection in rats [18-21]. Mp65p is a putative β-glucanase adhesin with one N- and multiple potential O-glycosylation sites, homologous to Scw10p of *S. cerevisiae*, a member of the GH17 glycosyl-hydrolase family [14,21,22]. Moreover, it contains a putative Kex2 peptidase (KR) site [23], where the protein is cleaved for secretion and an RGD motif that characterizes various proteins of eukaryotic organisms involved in adhesion mechanisms, as both adhesins and adhesin receptors [24,25]. Furthermore, we found that the *MP65 *gene can be used as a diagnostic marker for systemic *C. albicans *and non*-albicans *infections [26]. In another study [21], we described the construction of the *mp65Δ *mutants and some of their genetic traits and biological properties, demonstrating that Mp65p is required for hyphal morphogenesis and experimental pathogenicity. In the present study, we explored the role of Mp65p in depth, examining whether it is required for cell wall integrity, adhesion to host tissues and biofilm formation.

## Methods

### Microorganisms, media and growth conditions

The *C. albicans *strains used in this study are listed in Table 1. They were grown in YEPD (0.5% yeast extract, 1% peptone, and 2% glucose) or SDB (1% bacto-peptone, and 2% dextrose) media at 28°C, as described in the specific experiments. All media were solidified with 2% agar. Microbiological powders (yeast extract, peptone, and glucose) were obtained from Becton Dickinson (Becton Dickinson & Co., Sparks, MD). Laminarin (a linear β1,3-linked glucan backbone with occasional β1,6-linked branching), mannan, chitin (β-1,4-Nacetylglucosamine/β-1,4-N-acetylglucosamine-linked) and glucosamine were purchased from Sigma-Aldrich (St Louis, MO); pustulan (a β1,6-linked, linear glucan) was obtained from Calbiochem (La Jolla, CA); and β1, 3 glucanase Zymolyase 100T was obtained from Seikagaku Corporation (Tokyo, Japan).

**Table 1:** Strains used in this study

Nomenclature used in this study	Strain	Parent	Genotype	Reference
wild type	NGY152	CAI-4	as CAI-4 but *RPS1*/*rps*::CIp10	[56]

*mp65Δ *(hom)	RLVCA96	RLVCA35A	as CAI-4 but *MP65*::hisG/*MP65*::hisG, *RPS1*/*rps*:CIp10	[21]

revertant (rev)	RLVCA97	RLVCA35A	as CAI-4 but *MP65*::hisG/*MP65*::hisG, *RPS1*/*rps*:CIp10-*MP65*	[21]

### Sensitivity testing by microdilution method

To evaluate the sensitivity to cell wall-stressing agents, each *C. albicans *strain was initially grown for 24 h in YEPD; the cells were then washed with water, resuspended at OD_600 __nm _= 1, and inoculated in YEPD at OD_600 __nm _= 0.01; 95-ml volumes were then pipetted into microdilution plate wells. To these wells were added 5 ml of doubling dilutions of cell wall-stressing agents. The plates were incubated for 16 h at 30°C, and absorbance was read at 540 nm. All strains were tested in duplicate. The agents tested were: Congo red (Sigma, Milan, Italy; 100 mg/ml), calcofluor white (Sigma; 1000 mg/ml), SDS (Bio-Rad, Milan, Italy; 0.25%), caffeine (Sigma; 50 mM), and tunicamycin (Sigma; 100 mg/ml). The mentioned concentrations were the highest used to test each agent.

### Sensitivity testing by spotting in solid medium

To assess the susceptibility to specific cell wall-stressing agents, yeast cells were grown in YEPD, in agitation overnight (o.n.) at 28°C and then harvested, washed and re-suspended in sterile water. A sample containing 1.6 × 10^7 ^cells/ml and a series of 5-fold dilutions from the sample were prepared. Three μl of each dilution were spotted onto YEPD or YEPD buffered plates (buffered with 50 mM HEPES-NaOH pH 7.0, [4]), containing no additional chemicals (as control), Congo red (100 mg/ml in YEPD buffered plates), calcofluor white (100 mg/ml in YEPD buffered plates), SDS (0.025%), caffeine (10 mM), and tunicamycin (1.25 μg/ml). The plates were incubated for 24 h at 28°C.

### Sensitivity to Zymolyase

Sensitivity to Zymolyase was assayed as described previously [27]. Exponentially growing cells were adjusted to an OD_600 nm _value of 0.5 (approximately 2 × 10^7 ^cells/ml) in 10 mM Tris/HCl, pH 7.5, containing 25 μg/ml of Zymolyase 100T; the optical density decrease was monitored over a 140 min period.

### Morphology analysis

For morphological observations (inclusive of flocculation), the cells were grown at 28°C in YEPD in the absence or presence of Congo red and observed under a light microscope at 2, 6 and 24 h. The images were captured with Nikon Microphot-Fx and Arkon software and imported to Adobe Photoshop 7 (Adobe System Incorporated, San Jose, CA). Finally, the cropped images were assembled into figures using Canvas 9 (Deneba, Miami, FL). For the flocculation studies, following o.n. growth, the cultures were transferred to test tubes and incubated for 10 min. For scanning electron microscopy (SEM) observations, *C. albicans *cells were grown in YEPD in the absence or presence of Congo red (50 μg/ml) at 28°C for 2, 6 and 24 h. After centrifuging, the cells were washed twice in distilled water and fixed with 2.5% (v/v) glutaraldehyde in 0.1 M cacodylate buffer (pH 7.4) containing 2% (w/v) sucrose, for 20 min at room temperature (r.t.). After 3 washes in the same buffer, the cells were postfixed with 1% (w/v) OsO_4 _for 1 h, dehydrated through graded ethanol concentrations, critical point-dried in CO_2 _(CPD 030 Balzers device, Bal-Tec, Balzers) and gold coated by sputtering (SCD 040 Balzers device, Bal-Tec). The samples were examined with a Cambridge Stereoscan 360 scanning electron microscope (Cambridge Instruments, Cambridge, United Kingdom). For transmission electron microscopy (TEM), cells were prefixed with glutaraldehyde, as previously mentioned, then post-fixed with the OsO_4 _solution o.n., at 4°C. The cells were then dehydrated in acetone gradient and embedded in epoxy resin (Agar 100 resin, Agar Scientific Ltd, Stansted, UK), as per routine procedures. Ultrathin sections, obtained with an LKB ultramicrotome (LKB, Bromma, Sweden), were stained with uranyl acetate and lead citrate. These were examined with a Philips 208 transmission electron microscope (FEI Company, Eindhoven, Netherlands).

### Immuno-labelling studies in Electron Microscopy (EM)

For β-glucan localization in the post-embedding procedure, the ultrathin sections, obtained as described above, and collected on gold grids, were treated for 3 min with 0.5 mg of sodium borohydride per ml of ice-cold distilled water. After being washed in ice-cold distilled water (3 times, for 5 min) and in PBS containing 0.5% (w/v) bovine serum albumin, 0.05% Tween 20, and 5% fetal serum (3 times, 5 min each time), the sections were incubated with mAb 1E12 (diluted 1:10) o.n. at 4°C. After being washed at r.t. for 2 h by floating the grids on drops of PBS, the samples were labeled with rabbit anti-mouse immunoglobulin M (IgM) gold conjugate 10 nm (diluted 1:10; Sigma) and then washed in PBS buffer at r.t for 3 h. For negative control, the sections were incubated with IgM monoclonal antibody or with goat anti-mouse IgG-gold alone.

### Adhesion to buccal ephitelial cells (BEC)

Adhesion to buccal epithelial cells (BEC) was assayed as described previously [28]. Yeast cells were grown for 24 h at 28°C in Winge (0.3% yeast extract, 0.2% glucose), washed twice with PBS (0.02 M NaH_2_PO_4 _H_2_O, 0.02 M Na_2_HPO_4 _12H_2_O, 0.15 M NaCl, pH 7.4) and resuspended at 2 × 10^7 ^cells/ml in the same buffer by using a Bürker hemocytometer. One ml of yeast suspension was added to 10^5 ^BEC and incubated for 1 h at 37°C. The non-adhering fungal cells were washed off with 50 ml of PBS through a 12 μm polycarbonate filter. The filters were then gently smeared on glass slides, which were air-dried at r.t. o.n. stained with crystal violet (CV) and observed under a light microscope. The images were captured with Nikon Microphot-Fx and Arkon software at different magnifications, and

imported to Adobe Photoshop 7 (Adobe System incorporated, San Jose, CA) and then assembled into figures using Canvas 9 (Deneba, Miami, FL). Adherence was expressed as yeast cells adhering to 100 epithelial cells + standard error.

### Adhesion to Caco-2

The adhesion assay was set up in 24-well polystyrene plates as described previously [29], with only one modification: 2 × 10^2 ^cells in PBS (Phosphate Buffered Saline, Sigma) were added to each well.

### Biofilm formation and quantification

Cells were grown for 24 h at 28°C in YEPD broth. These were washed twice with sterile PBS (10 mM phosphate buffer, 2.7 mM potassium chloride, 137 mM sodium chloride, pH 7.4, Sigma), and resuspended in RPMI 1640 supplemented with morpholinepropanesulfonic acid (MOPS) at 1 × 10^6 ^cells/ml. The cell suspension (250 μl) was seeded in presterilized, polystyrene flat-bottom 24-well microtiter plates (Falcon, Becton Dickinson, NY, USA) and incubated for 48 h at 37°C. After biofilm formation, the medium was aspirated, and non-adherent cells were removed by washing the biofilms 3 times with 250 μl of sterile PBS [3,30]. The yeasts were quantified by the 2,3-bis (2-methoxy-4-nitro-5-sulfophenyl)-2H-tetrazolium-5-carboxanilide (XTT) reduction assay. The XTT (Sigma-Aldrich: 1 mg/ml in PBS) and menadione (Sigma: 0.1 M in acetone) solutions were prepared immediately before each assay. XTT solution was mixed with the menadione solution at a ratio of 1000:1 by volume; 250 μl of the XTT-menadione solution was then added to each well. The microtiter plates were then incubated in the dark for 1 h at 37°C. Following incubation, 250 μl of the XTT-menadione solution was recovered and centrifuged (to eliminate interference of cells with colorimetric readings); 100 μl of the solution was transferred to new wells, and the color change resulting from XTT reduction was measured at 490 nm with a microtiter plate reader (SpectraMax Plus microplate spectrophotometer; Molecular Devices, Ltd., Sunnyvale, CA). The absorbance values of the controls were then subtracted from the values of the test wells to eliminate spurious results due to background interference. Biofilm cultures were grown in triplicate, and each assay was performed 3 times. For the photographs, the biofilms were stained with CV [31] and the images captured with a Nikon Eclipse TE300 inverted microscope. For dry weight determinations, the biofilms were grown as described above and dried o.n. in a laminar flow hood. Three 24-well microtiter plates, for each C. albicans strain were used. The dry weight was given by the difference between the weight of dried plate containing biofilm and the same clean and sterile pre-weighed plate. The dry weight was expressed as the mean + S. D. of 3 plates.

### Quantitative Real-Time RT-PCR

The quantitative expression of different genes was determined by real-time reverse transcription (RT)-PCR starting from total RNA of *Candida *cells grown in YEPD o.n. at 28°C and then washed with DEPC treated water. Total RNA was extracted as previously described [32] and then treated with RNase-Free DNase (Roche) to remove traces of genomic DNA. The absence of DNA contamination was confirmed by a reverse transcription reaction using a control set of primers excluding the reverse transcriptase component from the cDNA reaction. Primer pairs for the target and reference *ACT1 *genes (Table 2) were designed using Beacon Designer software version 7.2.1 and synthesized by Primm (Milan, Italy). The first-strand cDNA synthesis from 1 μg of RNA was performed using QuantiTect Reverse Transcription Kit (Qiagen Hilden, Germany). In a total volume of 25 μl, iQ SYBR Green Supermix (Bio-Rad, Hercules, CA), 4 μl of first-strand cDNA reaction mixture, and 0.5 μM of primers were mixed. PCR was performed for samples in triplicate using the iCycler iQ Real-Time PCR detection system (Bio-Rad). A sampling program comprising of 95°C for 5 min, 40 cycles at 95°C for 45 s, and then at 58°C for 30 s was used. The amplification products were detected with SYBR Green, and the specificity of the amplification was confirmed by melting curve analysis. Bio-Rad iQ5 software was used to calculate CT values; the analysis of relative gene expression data was performed by the 2^-ΔΔCT ^method [33], with *ACT1 *as the reference gene.

**Table 2:** Oligonucleotides used in this study

Gene name	Oligonucleotide	5' to 3' sequence	Localization	
			**from/to**	**orf**
			
*MP65*	MP65f	TGTTGTTGTCACTATTGGTAATGG	126-149	19.1779
	MP65r	CGGCAGCAGAAGAAGAAGC	318-300	

*DDR48*	DDR48f	AACAACGACGACTCTTATGG	85-104	19.4082
	DDR48r	TGGAGGAACCGTAGGAATC	214-196	

*PHR1*	PHR1f	GTGTTGAACCAGTATTACCTTGAC	1321-1344	19.3829
	PHR1r	GGAAGATGCCTTACCAGTAGC	1461-1441	

*STP4*	STP4f	CCACATTATGAGCAAGAGTATAG	217-239	19.909
	STP4r	TACACAGACGAGGAAGCC	353-336	

*CHT2*	CHT2f	GCTACTACACAATCTACCACTAC	940-962	19.3895
	CHT2r	TTGAAGAAGAGGAGGAGGAAG	1096-1076	

*SOD5*	SOD5f	TTACAATGGAACCGTTAG	288-305	19.2060
	SOD5r	TAGGAGTCGTCATATTCA	401-384	

*ACT1*	ACT1f	CGATAACGGTTCTGGTATG	691-709	19.5007
	ACT1r	CCTTGATGTCTTGGTCTAC	786-768	

### Protein Extract and Western Analysis

To investigate if the cell wall integrity pathway was activated by the presence of Congo red, *C. albicans *cells were grown in YPD medium at 28°C, to mid-exponential phase, then treated with Congo red (50 μg/ml), 1.5 h before collection. The cells were then washed and resuspended in extraction buffer [100 mM Tris- HCl pH 7.5, 0.01% (w/v) SDS, 1 mM dithiothreitol, 10% (w/v) glycerol, protease inhibitor mixture (Roche Applied Science, Lewes, UK)] and then disrupted with glass beads. The lysate was centrifuged at 12 000 rpm for 10 min. The protein extracts were quantified using the Comassie protein assay reagent (Bio-Rad). One hundred and fifty μg of protein was separated on a 10% SDS-PAGE linear gel and then blotted to the nitrocellulose membrane. Before blocking, the equal loading was verified by MemCode ™ Reversible Protein Stain Kit (Pierce) together with the intensity of nonspecific bands. The membrane was then blocked in TBS plus 0.1% Tween 20 and 5 mg/ml dry milk (Carnation) at r.t. for 2 h. The anti-phospho-p44/42 MAPK (Thr^202^/Tyr^204^) antibody (New England Biolabs Inc., Hertfordshire, UK) was used to detect phosphorylated forms of Mkc1p and Cek1p MAPKs. The anti-MAPK antibody was used to reveal the total amount of Mkc1p. The anti-Kss1p polyclonal antibody (Santa Cruz Biotechnology), raised in rabbit against Kss1p of *S. cerevisiae*, was used to detect the total amount of Cek1p. The Act1p signal, obtained using the anti-Act1p antibody (SIGMA), was used as the loading control.

### Flow cytometry

To detect antigen expression, a suspension of 10^6^-10^7 ^yeast cells was fixed with 2% paraformaldehyde at r.t. for 30 min. After washing with ice-cold PBS, samples were incubated at 4°C for 30 min with mAb 1E12 diluted 1:100 and then with a goat anti-mouse IgM-fluorescein-conjugated antibody (Sigma) diluted 1:25. After washing, cells were immediately analyzed. Fluorescence was analyzed with FACScan flow cytometer (Becton Dickinson, Mountain View, CA) equipped with a 15 mW, 488 nm, air-cooled argon ion laser. FITC fluorescence was measured through a 530 nm band-pass filter and acquired in log mode. Negative controls were obtained by incubating samples with mouse IgM lambda (Sigma). The β-glucan content was expressed in arbitrary units (A.U.) and was calculated as the ratio of the labeled samples on the mean fluorescence channel (mfc) of the corresponding negative controls. The mfc was calculated by Cell Quest software (Becton Dickinson, Mountain View, CA).

### Cell wall components

The determination of the sugar monomers, after cell wall polysaccharides extraction with acid hydrolysis, was performed using HPLC with a Dionex Bio-LC system as previously described [34].

### Statistics

Differences in mean values of analytical determinations were assessed by the Student's t test, and significance was set at P < 0.05.

## Results

### Cell wall integrity

**Figure 1:**
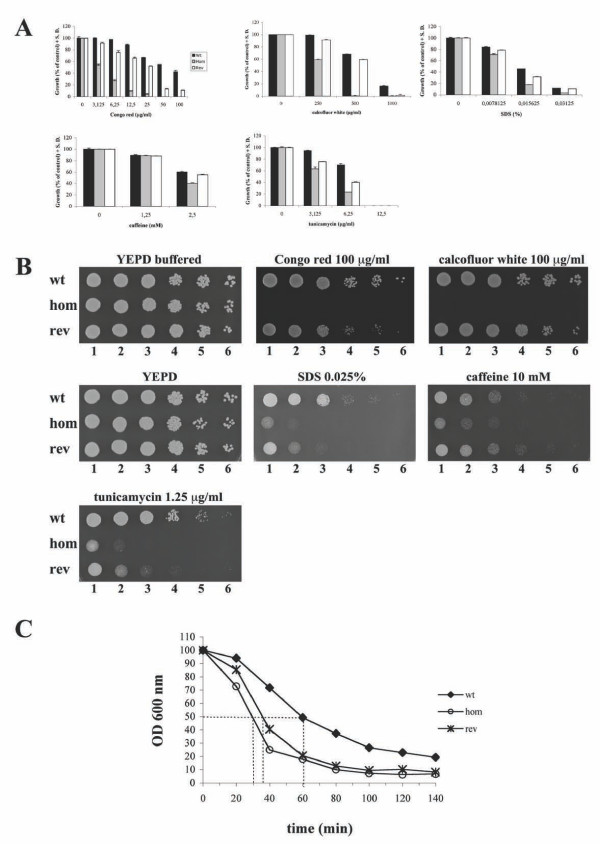
**Sensitivity of the *mp65Δ *mutant to different cell wall-perturbing and degrading agents**. (A) Microdilution sensitivity assay. The wild type (wt: black column), *mp65Δ *mutant (hom: grey column) and revertant (rev: white column) strains were quantitatively tested for sensitivity to different cell wall-perturbing agents using the microdilution method, as specified in the Methods section. Each column represents the mean of 3 experiments, with the bars representing standard deviations. (B) Solid medium spotting assay. The wild type (wt), *mp65Δ *mutant (hom) and revertant (rev) strains were tested by spotting decreasing cell counts on YEPD plates with or without cell wall-perturbing agents, as specified in the Methods section. Column 1 corresponds to the cell suspension and columns 2-6 correspond to 1:5 serial dilutions. (C) Sensitivity to Zymolyase. The wild type (wt), *mp65Δ *mutant (hom) and revertant (rev) strains were incubated in 10 mM Tris/HCl, pH 7.5, containing 25 μg/ml of Zymolyase 100T; the optical density decrease was monitored over a 140 min period.

To determine the effects of deleting the *MP65 *gene on the integrity of the cell wall, we tested the *mp65Δ *mutant for sensitivity to different agents whose effects have been associated with an altered cell wall. The sensitivity was measured by microdilution sensitivity and with solid medium spotting assays. In the first method, by performing dose-response experiments, the *mp65Δ *mutant was more sensitive than the wild type to the cell wall-perturbing agents Congo red and calcofluor white, which bind with glucan and chitin, interfering with their synthesis and cross-linking [35]. The *mp65Δ *mutant was also more sensitive than the wild type to SDS (a detergent that compromises the integrity of the cell membrane [36,37]), tunicamycin (a nucleoside antibiotic that inhibits N-linked glycosylation, affecting cell wall and secreted proteins [38-41]), and, though to a much lesser extent, caffeine (Figure 1A) (an inhibitor of cAMP phosphodiesterase, which effects the yeast cell surface [35,37,42]). In the second method, the data from single high-dose experiments (Figure 1B) confirmed the increased susceptibility of the *mp65Δ *mutant to all tested perturbing agents. The re-introduction of one copy of the *MP65 *gene (revertant strain) restored growth in the presence of all perturbing agents (totally or partially, depending on the perturbing agent and test conditions), demonstrating that the absence of this gene was responsible for the observed phenotype in a stress agent-dependent and gene-dosage dependent fashion.

To further assess the importance of Mp65p for cell wall assembly and integrity, we performed a cell wall digestion assay with a cell wall-corrupting β1,3-glucanase enzyme (Zymolyase 100 T) by measuring the half-life (the time required for a 50% decrease in the OD) of spheroplast lysis.

The *mp65Δ *mutant proved to be more sensitive to β-1,3-glucanase activity than the wild type and the revertant strains (30-min spheroplast half-life versus 60 and 37 min, respectively), indicating marked changes in the cell wall composition, organization or both, which could only in part be recovered by reintroduction of one copy of the *MP65 *gene (Figure 1C).

**Figure 2:**
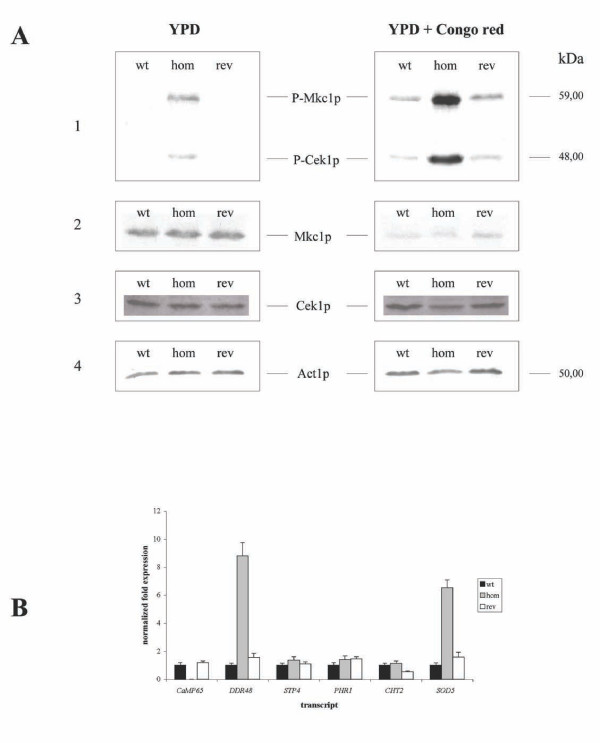
**Gene and protein expression in the *mp65Δ *mutant**. (A) Activation of the cell wall integrity. Activation of the cell wall integrity pathway was determined by Western blot analysis, as specified in the Methods section. The wild type (wt), *mp65Δ *mutant (hom) and revertant (rev) strains were grown in YEPD for 1.5 h at 28°C with or without Congo red (50 μg/ml). Protein extracts (150 μg) were loaded in each lane and analyzed with anti-p44/42 MAPK (panel 1), anti-MAPK (Panel 2), anti-Cek1p (Panel 3) and anti-Act1p (Panel 4) antibodies. (B) Cell wall damage response genes expression. Real-time PCR assays were conducted on RNA samples from wild type (wt), *mp65Δ *mutant (hom) and revertant (rev) strains. The reactions assayed the RNA levels of the *MP65*, *DDR48*, *PHR1*, *STP4*, *CHT2 *and *SOD5 genes*. The level of each RNA was normalized to the *ACT1 *RNA. The results are the means of 3 determinations. The bars indicate standard deviations.

The hypersensitivity of the *mp65Δ *mutant to cell wall-perturbing agents and the alterations in cell-wall organization (described below) led us to investigate whether the cell integrity pathway was activated in this mutant. Given that the induction of the cell integrity pathway resulted in the phosphorylation of the MAPK Mkc1p, we checked the activation of Mkc1p by Western blot with an anti-phospho-p44/42 MAPK (Thr^202^/Tyr^204^) antibody that binds to phosphorylated Mkc1p. As shown in Figure 2A, Panel 1, Mkc1p was activated in the *mp65Δ *mutant, whereas it was not activated in the wild type and revertant strains. For positive controls, the strains were stressed for 1.5 h with Congo red, whose cell wall-perturbing effect is known to induce Mkc1p phosphorylation. Also in this case there was activation of the cell integrity pathway. Using the mentioned antibody, an additional band, which is usually observed along with Mkc1p, and corresponds to the phosphorylated form of the MAP kinase Cek1p, was also detected (Figure 2A, Panel 1). The specificity of this antibody was ascertained by: i) the correspondence between the expected and observed band MW; ii) the disappearance of the 59 kDa band in an mkc1p mutant and its re-appearance in two different MKC1 reintegrant strains, as already demonstrated in previous studies [42,43]; iii) the barely detectable background in Western-blots; and iv) the different levels of expression of the examined proteins on the different samples. To rule out that the differences in the band appearance and intensity were due to changes in protein level rather than just their phosphorylated state, we performed a Western-blot analysis with anti-MAPK and anti-Kss1p antibodies, which revealed the total amount of Mkc1p and Cek1p, respectively (Figure 2A, Panels 2 and 3). Moreover, we assessed equal amounts of proteins before and after loading by Protein Assay (Bio-Rad) and by MemCode Reversible Protein Stain Kit (Pierce), as specified in the Methods section. The Act1p signal was used as an internal loading control (Figure 2A, Panel 4). Since the total level of Mkc1p did not change in the *mp65Δ *mutant compared to the wild type or revertant strains, the higher intensity of the band corresponding to the phosphorylated form of Mkc1p most likely resulted from hyperactivation of the upstream signaling pathway occurring in the *mp65Δ *mutant. Overall, we concluded that the *mp65Δ *mutant exhibited a constitutive activation of the Map kinases Mkc1 and Cek1, with a further increase after exposure to Congo red.

The above results suggest that the *mp65Δ *mutant may express cell wall damage response genes in the absence of exogenous cell wall-perturbing agents. We assayed the expression of the following five cell wall damage response genes: *DDR48*, *PHR1*, *STP4*, *CHT2 *and *SOD5 *[6,44-46]. Figure 2B shows that of the five genes mentioned only *DDR48 *and *SOD5 *had an altered expression in the *mp65Δ *mutant when compared to wild type and revertant strains. These findings suggest that the *MP65 *gene was required for the cell wall integrity and that *DDR48 *and *SOD5 *may be involved in the recovery of cell wall function when the *MP65 *gene is deleted. Overall, the *MP65 *mutation may have had a direct effect on the cell wall, given that *Mp65*p is a cell wall-located putative β1-3 glucanase enzyme [21], in addition to the indirect effects due to the altered expression of cell wall damage response genes.

### Morphological and biochemical properties of the mp65Δ mutant strain

**Figure 3:**
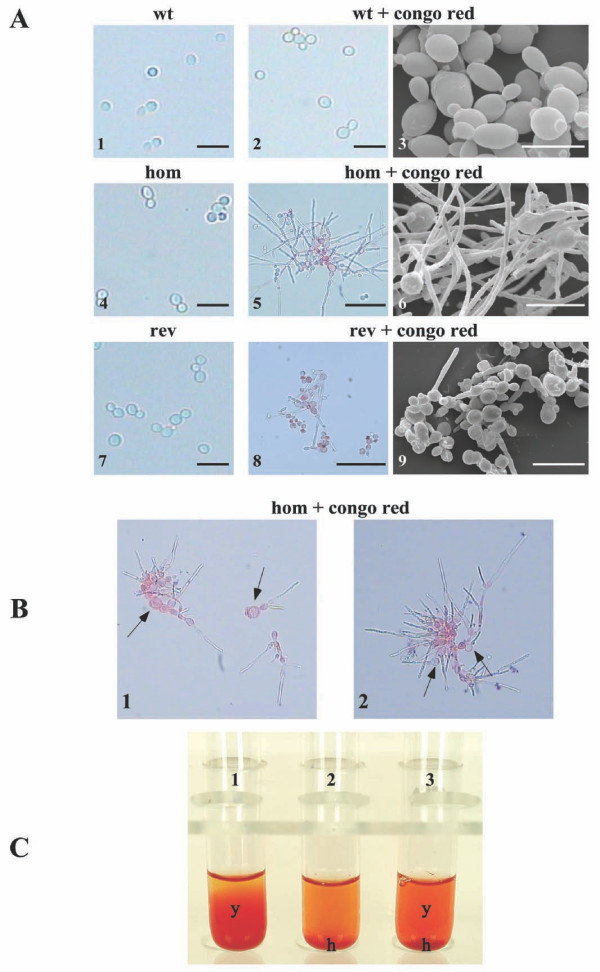
**Morphological analysis of the *mp65Δ *mutant**. (A) The wild type (wt), *mp65Δ *mutant (hom) and revertant (rev) strains were grown in YEPD for 24 h at 28°C with or without Congo red (50 μg/ml) and then observed under a light microscope and SEM, as described in the Methods section. The magnification bar corresponds to 15 μm (Panels 1, 2, 4, 6, 7 and 9), 5 μm (Panel 3), and 60 μm (Panels 5 and 8). (B) Pictures show swelling and clumping of the *mp65Δ *mutant cells after treatment with Congo red. (C) Flocculation analysis. Following o.n. growth, the cultures were transferred to test tubes and left to stand for 10 min. As shown, the filamentous cells (h) of the *mp65Δ *mutant precipitated to the bottom of the tube (hom: Tube 2). The yeast cells (y) of the wild type (wt: Tube 1) and revertant strains (rev: Tube 3) remained in suspension.

To study the cell-wall defects in more detail, we performed morphological, chemical, cytochemical and cytofluorimetric studies, mostly in cells responding to Congo red, which was the most intense perturbing agent. As shown in Figures 3A and 3B, Congo red-stressed *mp65Δ *mutant cells showed severe changes, such as swelling, clumping and formation of pseudohyphae and hyphae, compared with the wild type cells, which showed a normal yeast-shape appearance. The revertant strain showed an intermediate phenotype consisting predominantly of yeasts and some hyphae. Furthermore, the deletion of the *MP65 *gene affected flocculation: the *mp65Δ *mutant grown with Congo red showed marked flocs (Figure 3C).

**Figure 4:**
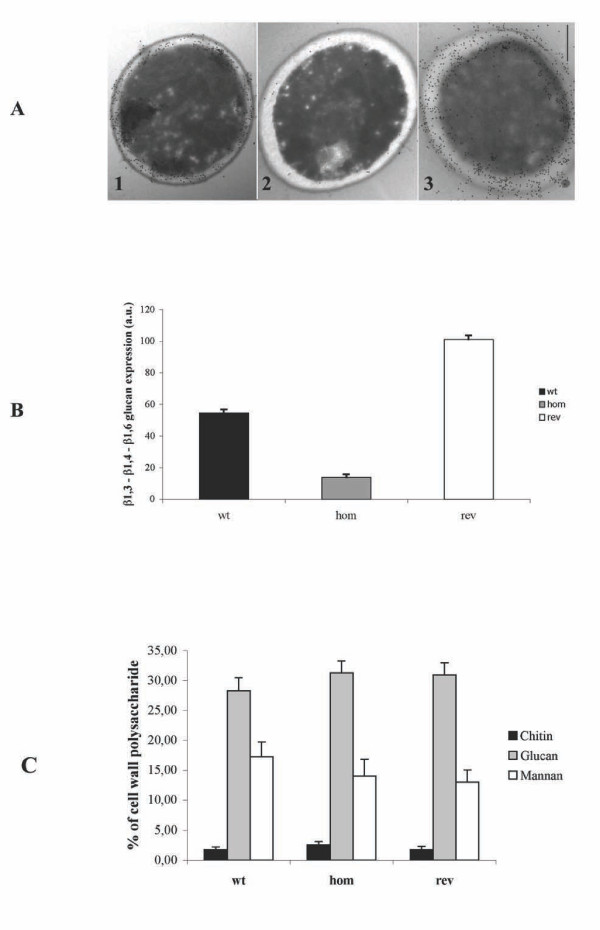
**Biochemical analysis of the *mp65Δ *mutant**. (A) Localization of β-glucan after glutaraldehyde fixation in the *mp65Δ *mutant, determined by Immunoelectron microscopy (IEM). This method of preparation avoids the use of osmium tetroxide and uranyl acetate and permits good cell preservation of the wild type (wt: Panel 1), *mp65Δ *mutant (hom: Panel 2) and revertant (rev: Panel 3) strains following post embedding labeling with the mAb 1E12 and followed by gold-labeled secondary antibody. The magnification bar corresponds to 0.5 μm. For more details, see the Methods section. (B) Expression of β-glucan in the *mp65Δ *mutant, as determined by flow cytometry. The β-glucan content is expressed in arbitrary units (A.U.) and was calculated as the ratio of the labeled samples on the mean fluorescence channel (mfc) of the corresponding negative controls. Each column represents the mean of 3 experiments, with the bars representing standard deviations (Mann-Whitney U test was used for statistical assessment). (C) Quantitative analysis of the cell wall sugar content by HPIC. The determination of the three principal cell wall polysaccharides (chitin, glucan and mannan) was performed, after extraction with acid hydrolysis, using HPIC with a Dionex Bio-LC system. The results are the mean of 3 independent experiments. The bars indicate standard deviations.

In the attempt to identify other indicators of cell wall changes, and given that *Mp65*p is a putative β-glucanase, we looked for the presence and distribution of β-glucan in the cell wall, using immunogold labeling and by FACS analysis. We used the monoclonal antibody 1E12, which recognizes all β-glucan types present in the *C. albicans *cell wall [7,47]. As shown in Figure 4A, the cells of the wild type strain had the expected intense and uniform labeling of the entire cell wall profile, with numerous gold particles randomly spanning cell wall layers. By contrast, the gold particles were much less numerous throughout the cell walls of the *mp65Δ *mutant, whereas the immunogold labeling was intense after re-introduction of the *MP65 *gene in the revertant strain. This suggested that the deposition of the β-glucan and its organization within the cell wall layers had changed in *mp65Δ *mutant strain, which was confirmed by the FACS analysis (Figure 4B).

We also investigated the possible chemical changes in the cell wall composition. As previously demonstrated in *Saccharomyces cerevisiae *(*fks1, mnn9, gas1, kre6, knr4, and chs3 *strains) [34] and *C. albicans *mutants (*kre5, crh*) [43,48,49], the defective expression in the genes implicated in cell wall biogenesis and regulation may also result in dramatic changes in the chemical composition of the cell wall. Hence, we measured the amount of main cell wall polysaccharide components (i.e., mannan, glucan and chitin). The comparison of the *mp65Δ *mutant with wild type indicated no statistically significant differences in any of these components (Figure 4C). However, there was a trend of an increase in chitin content in the *mp65Δ *mutant compared to the wild type cells (2.56 ± 0.57 vs. 1.75 ± 0.45: these values are the mean percentage distribution of chitin of 3 independent experiments expressed as mean + S.D.).

### Adherence and biofilm formation

**Figure 5:**
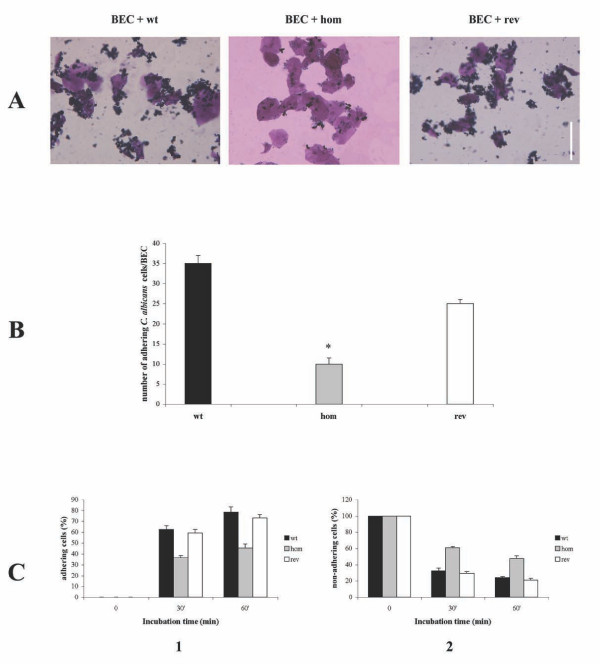
**Adhesion analysis of the *mp65Δ *mutant**. (A) Adhesion of the *mp65Δ *mutant to BEC. Representative fields randomly selected showing the interaction between yeast cells [wild type (wt), *mp65Δ *mutant (hom) and revertant (rev) strains] and BEC after 1 h of incubation at 37°C. The magnification bar corresponds to 100 μm. See the Methods section for more details. (B) Adhesion assay data. Histograms showing the adherence of the wild type (wt: black column), *mp65*Δ mutant (hom: grey column) and revertant (rev: white column) strains to BEC. The bars indicate the standard errors. Significant differences from wild type adhesion (P < 0.05) are indicated by asterisks. (C) Adhesion of the *mp65Δ *mutant to Caco-2 cell monolayers. Recovery of *Candida *cells [wild type (wt: black column), *mp65Δ *mutant (hom: grey column) and revertant (rev: white column) strains] at different time points (30 and 60 min) of incubation with Caco-2 cells. Adherent cells recovered after thorough washing out of the microplate (Panel 1). Non-adherent cells recovered from the supernatant (Panel 2). The results are the mean of 3 independent experiments. The bars indicate the standard deviations.

To examine the differences between wild type and *mp65Δ *mutant strains in their adherence to epithelial cells, we used two model cell systems: the exfoliated human buccal epithelial cells (BEC) and the Caco-2 cell monolayers. In the first system (visual assay of stained cells), 2 × 10^7 ^cells of wild type and *mp65Δ *mutant strains were incubated with 10^5 ^BEC, and the adherence was expressed as the number of yeast cells adhering to 100 epithelial cells ± standard error. The *mp65Δ *mutant showed significantly reduced adherence to BEC (Figures 5 A and 5B), whereas the revertant strain partially regained the ability to adhere to BEC, reaching a level similar to that of the wild type (*C. albicans *cells/BEC mean ± S.E.; wild type: 35 ± 2.0 vs. *mp65Δ *mutant: 10 ± 1.5 vs. revertant: 25 ± 1.0; P < 0.05). In the second system, the number of *C. albicans *cells adhering to the surface and those remaining in the supernatant were analyzed in a time-dependent manner (Figure 5C). Adhesion of the wild type cells to Caco-2 cells was rapid and efficient: after 30 min, about 65% of the cells recovered had adhered to the Caco-2 cell monolayers, whereas only 35% were recovered from the supernatant. After 60 min the percentage of adhering cells increased to 75%, whereas the percentage of cells in the supernatant decreased to 25%. The *mp65Δ *mutant cells showed significantly reduced adhesion to the Caco-2 cells: after 30 and 60 min, the percentage of adhering cells was 38% and 43% respectively, whereas the percentage of non-adhering cells was 62% and 57% respectively. In the revertant cells, the efficiency and kinetics of adhesion were similar to those in the wild type.

**Figure 6:**
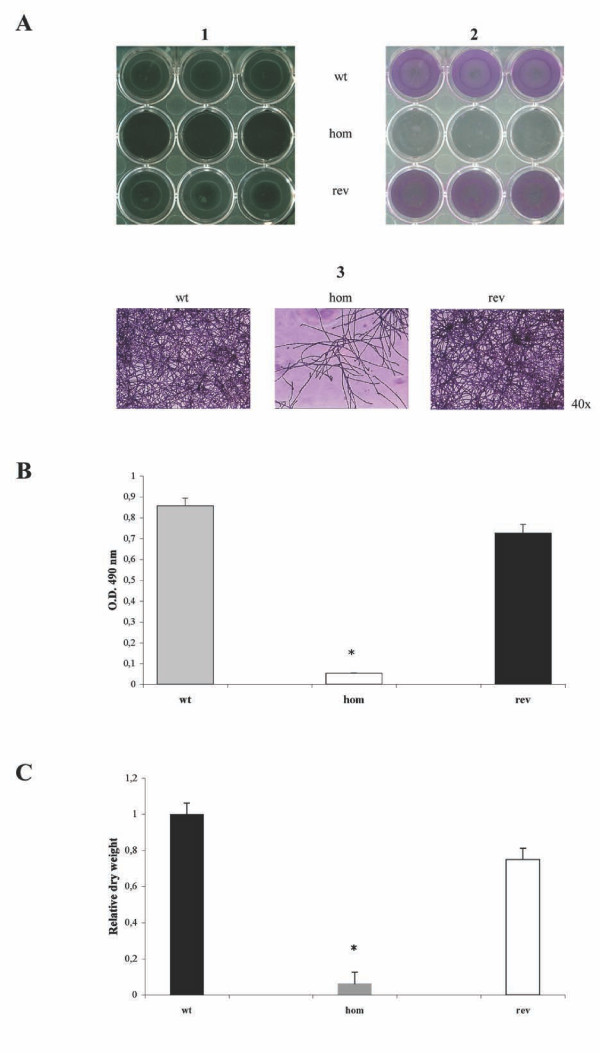
**Biofilm analysis of the *mp65Δ *mutant**. (A) CV staining. Equal numbers of cells from the wild type (wt), *mp65Δ *mutant (hom) and revertant (rev) strains were suspended in 250 μl of RPMI medium and incubated in 24-well plates for 48 h at 37°C. Non-adherent cells were then removed by washing, and adherent cells were stained with CV. The biofilms were visualized before (Panel 1) and after (Panel 2) staining and then captured by using either a Gel Doc system (Bio-Rad), or using an inverted microscope at 40x magnification (Panel 3). (B) XTT assay. The colorimetric XTT assay, which determines the metabolic activity of the cells, was used to quantify the biofilms of the wild type (wt: grey column), *mp65Δ *mutant (hom: white column) and revertant (rev: black column) strains. Each result is the mean of 3 independent experiments (P≤ 0.05, Student's *t*-test, two-tailed, for comparison of dry weight of hom vs. wt and rev strains; error bars represent standard deviations). (C) Dry weight determination. The dry weight determination, which measures the total biomass of the cells, was used to quantify the biofilms of the wild type (wt: black column), *mp65Δ *mutant (hom: grey column) and revertant (rev: white column) strains. Results were normalized to wt, which was taken as 100%. Each result is the mean of 3 independent experiments (P ≤ 0.05, Student's *t*-test, two-tailed, for comparison of dry weight of hom versus wt and rev strains; error bars represent standard deviations.

To determine the effects of the absence of the *MP65 gene *on biofilm formation, we performed two quantitative *in vitro *assays (dry weight and XTT), which characterize total and living biomass, respectively. As shown in Figures 6 B and 6C, the *mp65Δ *mutant displayed a severe defect in biofilm formation compared to the wild type strain, and the introduction of a single wild type *MP65 *allele in the revertant strain substantially salvaged the defect. These results were confirmed by observation of the biofilms before and after staining with CV, a semi-quantitative colorimetric assay that does not differentiate between live and dead cells (Figure 6A). Similar results were obtained when the biofilms were grown in spider medium (Additional file 1, Figure S1 and other data not shown).

## Discussion

The cell wall is a dynamic structure that is remodeled when fungal cells are exposed to severe stress conditions, including hyphal growth, mutations of genes coding for cell wall components, and host immune responses [34]. This remodeling leads to a reorganization of the cell wall architecture following the activation of different cell-wall compensatory mechanisms [50]. The 65-kDa mannoprotein (*Mp65*p) of *C. albicans *was previously shown to be a major target of anti-*Candida *immune responses in humans [15-17] and, more recently, a putative β-glucanase adhesin which plays a critical role in hyphal formation and virulence of this fungus [18-21].

In light of these findings, we have now specifically addressed the role of *Mp65*p in cell wall biogenesis and integrity, as well as the adherence to epithelial cells and biofilm formation.

Also based on previous work performed with scw4scw10 mutants of *S. cerevisiae *[51] and *pkc1*, *mkc1*, *hog1 *[42], *pmr1 *[35], *och1 *[38], *sun41 *[5] and *crh *mutants of *C. albicans *[43], we first examined the sensitivity of the *mp65Δ *mutant to a range of cell wall-perturbing agents to determine the effects of the *MP65 *gene deletion on the integrity of the cell wall.

Our data show that *Mp65*p plays an important role in membrane/cell wall stability. This was evident from: *i*) the increased sensitivity of the *mp65Δ *mutant to a number of agents whose effects have been associated with altered cell wall; *ii*) the constitutive activation of the cell wall integrity pathway in the mutant; *iii*) the increased expression in the mutant, in the absence of stressing agents, of *DDR48 *and *SOD5*, two cell wall damage response genes which code for, respectively, a cell-wall protein and an antioxidant enzyme [44-46].

Interestingly, the cell wall defects consequential to the *MP65 *gene deletion did not bring about gross detectable changes in the cell wall chemistry, as seen in other mutants of β-glucanase enzyme families [50,52]. While further investigations are needed to detect small chemical changes, which are likely to occur in the mutant cell wall, we believe that the *MP65 *gene deletion may mostly affect cell wall organization, with associated remodeling of its main polymeric constituents. This interpretation is supported by the comparable contents of all the 3 cell wall polysaccharides (mannan, glucan and chitin), which overall accounted for more than 95% of the cell wall dry weight, and by the rather marked differences in β-glucan expression, zymolyase sensitivity and morphological changes on the other. In particular, the disposition of β-glucan appears to be affected in the *mp65Δ *mutant, which displays a much lower reactivity than the wild type cell, as detected by an antibody which recognizes both β-1,3 and β-1,6 glucan configurations. This would suggest that β glucan is much less accessible to the antibody in the *mp65Δ *mutant than in the wild type strain. This lower antibody accessibility to the target may modulate immune responses to the pathogen, in view of the critical role exerted by β-glucan polysaccharide in fungal recognition by the immune system [53].

Notably, the re-integration of one *MP65 *gene copy in the revertant strain did not induce a full recovery of the lost or decreased function of the *mp65Δ *mutant. This is in line with the repeatedly observed gene dosage effects in *C. albicans *[54].

Some β-glucanase mutants have been shown to be endowed with low pathogenicity potential which is not entirely attributable to their inability to make tissue invasive hyphae [22,50]. The adherence to host tissues or to abiotic surfaces is an important attribute of *Candida *that is positively correlated with pathogenicity [54]. In *C. albicans *and *C. glabrata*, but also in the less pathogenic yeast *S. cerevisiae*, multiple adhesion proteins (known as "adhesins", "flocculins" or "agglutinins") have been identified, such as Als family proteins, Hwp1, Eap1 in *C. albicans *and Epa proteins in *C. glabrata*. These proteins provide these organisms with a variety of adherence properties, such as their interactions with other cells (during mating) and with abiotic surfaces and host tissues. *Mp65*p is a putative β-glucanase adhesin, which is critical to *C. albicans *adherence to an abiotic surface [21]. In this study, we explored whether the adherence to epithelial cells was also affected in the *mp65Δ *mutant. We thus compared the ability of the wild type and the *mp65Δ *mutant strains to adhere to BEC and Caco-2 cell monolayers by using two *in vitro *adhesion assays. In both assays, the *mp65Δ *mutant consistently displayed a significant decrease in adherence. These findings, together with the capacity of an anti-*Mp65*p serum to inhibit almost totally the adherence to the plastic by the wild type strain [21], highlights the more exstensive role of Mp65p as an adhesin, in that its adhesion is not limited to inert surfaces. Nevertheless, the decreased adherence of the *mp65Δ *mutant could also be indirectly due to the suggested alteration in cell wall organization, with a possible decreased cell surface expression of other *C. albicans *adhesins, such as those previously mentioned.

Biofilms are typically found on medical devices, such as catheter surfaces, and they have attracted attention because of their persistence and resistance to antifungals [3,30]. Given that biofilm formation begins with surface adherence and that *mp65Δ *mutant loses adherence to the polystyrene plates, as demonstrated in our previous paper [21], we also investigated whether the ability of the *mp65Δ *mutant in forming biofilms had altered. As consistently shown by our data, the *mp65Δ *mutant displayed a strongly defective biofilm formation, in contrast to wild type that produced abundant biofilm.

## Conclusions

The findings reported in the current paper significantly extend beyond the previously reported role of *Mp65*p in hyphal cell wall biogenesis and actually confirm that morphogenesis and cell wall remodeling are intimately related issues [22,50,55]. The knock-out of the *MP65 *gene affects biological properties that are of potential relevance for candidiasis. Together with the defective hyphal morphogenesis [21], these findings provide some further functional correlates to the previously demonstrated loss of invasive and mucosal pathogenicity by the *mp65Δ *null mutant. Overall, the *MP65 *gene appears to play a role in cell wall structure and stability which, by still unknown mechanisms, are translated into fungal virulence. For all of the discussed reasons, and with the previously reported evidence of Mp65p being a major target of host immune response to C. albicans [12], this protein remains an interesting potential target for therapeutic or immunotherapeutic interventions.

## Authors' contributions

SS conceived the study, its design and coordination, drafted the manuscript and performed sensitivity testing, morphology analysis, adhesion to BEC and Caco-2, biofilm formation, quantitative Real-Time RT-PCR, protein extract and Western-blot analysis.

AS participated in the design of the study drafted the manuscript and carried out FACS and biofilm analysis. SA, FM and AG helped SS in the experimental studies.

MC and NM conducted the immuno-labelling studies in EM, the morphology analysis by TEM and generated Caco-2 cell monolayers for adhesion studies. SM performed the HPLC analysis. FDB provided the funds and helped SS in the experimental planning. All authors read and approved the final manuscript.

## Supplementary Material

Additional file 1 **Figure S1: Biofilm analysis of the *mp65Δ *mutant in Spider medium**. Cells of the wild type (wt), *mp65Δ *mutant (hom) and revertant (rev) strains were visualized before (Panel 1) and after (Panel 2) staining and then captured by using Gel Doc system (Bio-Rad). Click here for file
