# Supplementary figures and images for: The MP65 gene is required for cell wall integrity, adherence to epithelial cells and biofilm formation in Candida albicans
Source: BMC Microbiol. 2011 May 16;11:106. doi: 10.1186/1471-2180-11-106 (PMC3113926; doi:10.1186/1471-2180-11-106)

Before staining

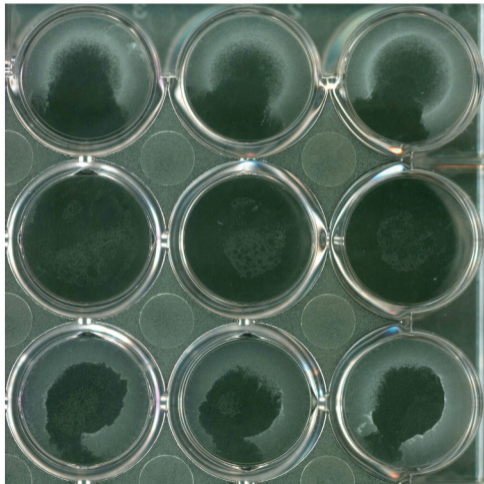

wt

hom

rev

After staining

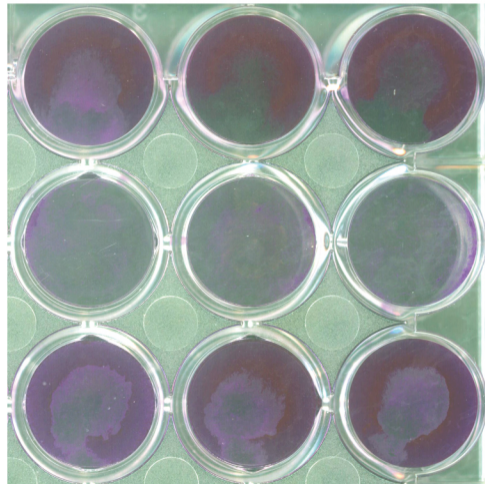

Supplement: Additional file 1 — Figure S1: Biofilm analysis of the mp65Δ mutant in Spider medium. Cells of the wild type (wt), mp65Δ mutant (hom) and revertant (rev) strains were visualized before (Panel 1) and after (Panel 2) staining and then captured by using Gel Doc system (Bio-Rad). [file 1471-2180-11-106-S1.PDF]
